# Risk-Adapted Surveillance in Borderline Ovarian Tumours (BOTs): The “Barts” Evidence-Based Framework

**DOI:** 10.3390/cancers18172764

**Published:** 2026-08-26

**Authors:** Sofia Lekka, Shaun Haran, Nadia Amel Seksaf, Iteeka Arora, Arjun Jeyarajah, Saurabh Phadnis, Alexandra Lawrence, Elly Brockbank, Ranjit Manchanda, Michail Sideris

**Affiliations:** 1 Department of Gynaecological Oncology, Royal London Hospital, Barts Health NHS Trust, London E1 1FR, UK; lekka.par.sofia@gmail.com (S.L.); shaunharan@nhs.net (S.H.); n.a.seksaf@smd22.qmul.ac.uk (N.A.S.); iteeka.arora@nhs.net (I.A.); arjun.jeyarajah@nhs.net (A.J.); s.phadnis@nhs.net (S.P.); alexandra.lawrence5@nhs.net (A.L.); elly.brockbank@nhs.net (E.B.); r.manchanda@qmul.ac.uk (R.M.); 2 Centre for Cancer Screening, Prevention & Early Diagnosis, Wolfson Institute of Population Health, Queen Mary University of London, London E1 4NS, UK; 3 Department of Health Services Research, Faculty of Public Health & Policy, London School of Hygiene & Tropical Medicine, London WC1H 9SH, UK

**Keywords:** borderline ovarian tumour, recurrence patterns, risk-adapted surveillance, patient-initiated follow-up, malignant transformation

## Abstract

**Background/Objectives:** Borderline ovarian tumours (BOTs) are distinct epithelial neoplasms with excellent survival but variable recurrence, including late relapse and occasional malignant transformation. Follow-up strategies remain inconsistent internationally, with no standardised, risk-adapted framework. We aimed to synthesise the evidence on BOT recurrence patterns, risk factors and surveillance strategies, and to propose a structured, risk-adapted surveillance framework. **Methods:** This is a narrative expert synthesis rather than a systematic review. Three evidence sources were combined: our previously published comprehensive review of BOTs, our recent meta-analysis of recurrence and malignant transformation, and an appraisal of contemporary international guidelines. Clinicopathological and surgical determinants of recurrence were then mapped onto the available follow-up tools through a three-step approach, and the resulting risk strata, surveillance intervals and total follow-up duration were agreed on by consensus within a single tertiary gynaecological oncology centre (the “Barts Framework”). **Results:** Recurrence occurs in 3–10% of patients, with up to one-third arising beyond five years. Key predictors include fertility-sparing surgery (particularly cystectomy), incomplete staging, advanced stage, residual disease, and adverse histological features such as micropapillary/cribriform architecture and invasive implants. Three targets for surveillance were identified: early detection of recurrence, detection of malignant transformation, and optimisation of fertility. Transvaginal ultrasound emerged as the cornerstone modality, with cross-sectional imaging and tumour markers applied selectively. Four risk strata were derived, each specifying follow-up intensity, modality, total duration and care setting. Low-risk patients can be managed in decentralised settings (gynaecological units), whereas high-risk groups warrant specialist oversight (gynaecological oncology centres). Patient-initiated follow-up is incorporated to minimise unnecessary interventions. **Conclusions:** This risk-adapted approach provides a scalable framework to standardise BOT surveillance whilst reducing overuse and maintaining oncological safety. The framework is consensus-based and single-institution in origin, and prospective external validation with long-term outcome data is required before wider adoption. Integration of molecular stratification and artificial-intelligence-assisted imaging represents a key future direction.

## Simple Summary

Borderline ovarian tumours are growths of the ovary that sit between benign cysts and ovarian cancer. Almost all women diagnosed with one will live a normal lifespan, yet the tumour can come back many years after the original operation and, in a small number of women, it can turn into a cancer. Women therefore need to be monitored after surgery. At present there is no agreement on how often they should be seen, which scans or blood tests they need, or whether this should happen in a local hospital or a specialist cancer centre. We brought together the published evidence to identify which women are more likely to have their tumours return. We then used this to design a practical monitoring plan that places each woman into one of four risk groups, and states for each group how often she should be seen, which tests she should have, for how long, and where. Women at low risk are spared unnecessary appointments and scans, while those at higher risk are followed more closely by specialists. The plan is a pilot proposal based on the current evidence and expert agreement. It will be tested prospectively across several hospitals before its final implementation.

## 1. Introduction

Borderline ovarian tumours (BOTs) are a heterogenous group of neoplasms characterised by increased epithelial proliferation and cytologic atypia compared to benign tumours [1,2,3]. Contrary to ovarian cancer (OC), they do not exert infiltrative destructive growth or stromal invasion [4]. BOTs comprise 15–20% of all epithelial ovarian tumours and hold an annual incidence of 1.8–4.8 per 100,000 women [5]. In the 5th edition of the WHO Classification of Female Genital Tumours (2020), these lesions are designated as atypical proliferative serous tumours and atypical proliferative mucinous tumours, with “serous/mucinous borderline tumour” retained as an accepted synonym [6]. Every type of surface epithelial cell has been reported to be the origin for BOTs, with serous (53.3%) and mucinous (42.5%) being by far the most common [7].

Serous BOTs (sBOTs) often demonstrate typical hierarchical branching patterns lined by cells that show low-grade nuclear atypia. Microinvasion is defined as invasion less than 5 mm in greatest dimension. The most common pattern includes clusters of cells or single cells with abundant eosinophilic cytoplasm (cytologically similar to the non-invasive component) invading the stroma, typically surrounded by a clear space [8]. Microinvasion is seen more commonly in pregnant patients but the presence of microinvasion does not alter the outcome [4]. Evidence suggests that women with Stage I disease have the same oncological outcomes irrespective of microinvasion. “Non-invasive implants” refers to papillary proliferations of atypical serous cells that involve the surface of the tissues or are present within the peritoneal/omental invaginations but do not the invade the underlying tissue. If destructive stromal invasion is noted, then this is defined as an invasive implant and the term extra-ovarian low-grade serous carcinoma (LGSOC) is appended. Expanded criteria have been proposed for implants that lack morphological evidence of tissue invasion but are nevertheless considered to be carrying adverse prognostic significance. These criteria include micropapillary architecture and solid nests of cells surrounded by cleft-like spaces. However, the validity of these features as predictors of clinical outcome remains to be established in larger studies, and currently we do not diagnose invasive implants in the absence of obvious tissue invasion [9].

Mucinous BOTs (mBOTs) typically present as large unilateral masses that are confined to the ovary. Peritoneal implants are rarely, if ever, convincingly documented in association with mBOTs [10,11]. Historical reports of mBOTs with pseudomyxoma peritonei are now generally attributed to a synchronous or occult appendiceal primary rather than to ovarian origin, and this possibility should be excluded before extra-ovarian mucinous disease is ascribed to the ovary. MBOTs can be heterogeneous, exhibiting additional features focally indicating progression. These features include intraepithelial carcinoma, microinvasion, and mural nodules. Intraepithelial carcinoma has generally been reported to carry a favourable prognosis, although the supporting series are small and retrospective and a modest excess risk of ovarian recurrence cannot be excluded; mural nodules are heterogeneous, and those containing anaplastic carcinoma or sarcomatous elements have been associated with adverse outcomes. Both features should therefore prompt careful pathological review rather than being assumed to be uniformly benign. Similar to sBOTs, microinvasion does not appear to adversely affect prognosis in the available evidence [10].

### 1.1. Recurrence of BOTs

Two-thirds of reported recurrence occur within the first five years following primary surgery. However, BOTs can be associated with late recurrence patterns, with cases reported beyond 10–15 years [7,12]. Late recurrence is a defining feature of BOT biology and supports the need for prolonged follow-up beyond the timeframe commonly used for invasive ovarian cancers. Median follow-up in most studies which report on recurrence is approximately five years, although some cohorts demonstrate recurrences after much longer intervals [13], with overall recurrence rates being reported between 3 and 10% [12]. In the systematic overview by du Bois et al., 37% of recurrences were diagnosed within the first 2 years, 31% between years 2 and 5, and 32% after year 5, with approximately 10% occurring beyond 10 years [14]. Treatment with cystectomy is associated with a 31% recurrence rate [7]. Recurrence is higher after conservative surgery (10 to 20% vs. approximately 5% for radical surgery, i.e., bilateral salpingo-oophorectomy, with or without hysterectomy and total omentectomy [15]). Nevertheless, this higher recurrence rate is not translated to a higher mortality [16]. Patients with extra-ovarian disease are faced with recurrence in up to 25% of cases. SBOTs with micropapillary and cribriform architecture have a greater association with extra-ovarian disease and a higher incidence of recurrence and death from disease than typical sBOTs [17]. Our recent meta-analysis summarises the most updated recurrence figures [17,18].

### 1.2. Patterns of Recurrence

Most recurrences remain BOTs; 20% of patients diagnosed with recurrence will have invasive ovarian cancer [12]. Malignant transformation happens more frequently (two-thirds) in women more than 40 years old [12,16,19]. Typical recurrence patterns include the ovary (most common site of relapse), particularly after cystectomy or unilateral ovarian preservation [1,3,13]. Peritoneal recurrence is almost exclusively associated with sBOTs and may manifest as peritoneal implants. Malignant transformation is rare but clinically significant and usually occurs as LGSOC in the context of sBOT recurrence. Our recent meta-analysis [13] evidence suggests malignant transformation occurs in approximately 1% of cases overall but increases substantially to 22% in recurrent BOT cases. Rare instances of sBOTs transforming to high-grade carcinoma are documented. Such high-grade transformation is associated with poor prognosis [20,21,22].

### 1.3. Risk Factors for Recurrence

Several clinicopathological factors are consistently associated with increased risk of recurrence (Table 1). Fertility-sparing surgery (FSS), and especially cystectomy rather than oophorectomy, is a recognised factor for higher recurrence rates (cystectomy ~31–41% vs. oophorectomy ~9–13% risk of recurrence) [9,10,12,15,19], consistent with the direction of effect reported in Section 1.1 and summarised in Table 1. Histopathological elements, including sBOTs with micropapillary and cribriform architecture, have a greater association with extra-ovarian disease and a higher incidence of recurrence as well as disease-related deaths compared to the typical sBOTs [17]. Two large retrospective series of women with BOTs showed that higher stage, incomplete staging, residual tumour, and FSS were independent prognostic factors for recurrence [9,23,24]. Invasive peritoneal implants have also been established as risk factors for recurrent or persistent disease [3,9]. Other factors associated include older age at diagnosis (40 years old and above), bilateral ovarian involvement, large tumour size, and fertility-sparing surgery [3,9]. Histology also influences recurrence patterns, with sBOTs more likely to recur as peritoneal disease whereas mBOTs are more likely to recur within the ovary, with greater risk of progression to invasive mucinous carcinoma when malignant transformation occurs [3,9]. The principle that recurrence risk after fertility-preserving gynaecological surgery is driven by a reproducible set of clinicopathological determinants, and that these determinants should govern the duration and intensity of surveillance rather than a fixed protocol, is well established for other benign and borderline gynaecological pathologies. A recent meta-analysis of recurrence after laparoscopic myomectomy provides a close methodological parallel [25].

### 1.4. Hysterectomy in BOTs

The role of hysterectomy in premenopausal women BOTs remains a grey area. In early-stage disease with no adverse histopathological risk factors or extra-ovarian disease, it seems to add no prognostic benefit. Specifically, where bilateral salpingo-oophorectomy has already been performed, the uterus is rarely involved by BOTs and completion hysterectomy confers little additional oncological benefit; it is therefore not routinely required [26,27]. The principal rationale for completion surgery applies after fertility-sparing surgery, where the preserved ovary is the commonest site of recurrence: removal of residual ovarian tissue (with or without hysterectomy) once the family is complete reduces the risk of recurrence. In postmenopausal women undergoing BOT surgery, a single multicentre retrospective study has reported outcomes favouring concomitant hysterectomy [28]; however, no survival benefit has been demonstrated. Hence, we should discuss hysterectomy individually, taking into account operative risk, co-existing uterine pathology and patient preference, explicitly underlining the higher risk of uterine involvement in the postmenopausal group of women.

### 1.5. Evidence Gap and Scope to Propose a Follow-Up Framework

One-third of patients with BOTs are younger than 40 years old with incomplete family plans, therefore necessitating the consideration of fertility-sparing approaches [12]. Evidence demonstrates long-term (late) recurrence potential which varies depending on histopathological (tumour-related) features, surgical staging approach (fertility vs. radical), including spillage, and residual tumour or patient’s age [13].

On these grounds, most groups agree on long-term follow-up, but a framework recommendation on follow-up structure, including scope, intensity (timeline), optimal imaging modality with or without tumour markers (TMs) or location (cancer unit or tertiary centre) is yet to be established. Evidence synthesis from published guidelines demonstrates considerable variation in follow-up protocols internationally, which can result in unnecessary burden on each healthcare system to deliver adequately frequent and imaging-based follow-up visits. On the other hand, evidence from breast cancer surveillance programmes suggests that offering intensive follow-up to low-risk women may provoke anxiety in some individuals [29]. To this end, we synthesised the available published evidence in order to propose a risk-adapted surveillance framework. The specific aims of this study were: (i) to summarise the evidence on the rate, timing, pattern and determinants of recurrence in BOTs; (ii) to appraise the tools available to deliver surveillance, namely clinical review, imaging modality, tumour markers and care setting; and (iii) to integrate these into a four-stratum, risk-adapted follow-up framework that specifies, for each stratum, the intensity, modality, total duration and location of follow-up.

## 2. Materials and Methods

In a previously published comprehensive review [3], we summarised the available evidence on several thematic axes around BOTs, including follow-up strategies. This included reviewing published studies, as well as scientific societies’ guidelines. We also performed a recent meta-analysis to calculate pooled recurrence risks across different surgical approaches [13]. Furthermore, we have reviewed molecular and histopathological features and their potential influence on recurrence potential and patterns [1]. In this study, we synthesised and expanded upon existing knowledge of BOT biology, identified recognised risk factors for recurrence, examined recurrence timelines and patterns across available follow-up data, and reviewed globally accepted surveillance strategies to propose an evidence-based, risk-adapted follow-up framework. We adopted a three-step approach:

- Step 1: Based on our previous work [1,3,13], we provide a succinct summary of background knowledge on factors influencing follow-up decision (section prior to Methods).
- Step 2: We summarise the reported scope of follow-up, the available tools to deliver it, including imaging modalities and tumour markers (TMs), as well as the potential location (infrastructure, i.e., cancer unit or tertiary centre).
- Step 3: Based on step 1, we conceptualised four potential “risk-strata”; importing the available follow-up tools (step 2) and reported follow-up patterns (timing/frequency) primarily based on several scientific bodies’ guidelines, we conclude with a risk-adapted follow-up framework (The “Barts Framework”).

### 2.1. Study Design, Evidence Sources and Appraisal

This work is a narrative expert synthesis rather than a systematic review and should be interpreted as such: no PRISMA-compliant search, duplicate screening or formal risk-of-bias assessment was undertaken for the present manuscript. The underlying evidence base was assembled in our two previous publications—a comprehensive review of BOTs [3] and a systematic review and meta-analysis of recurrence and malignant transformation [13]—in which the search strategies, eligibility criteria and appraisal methods are reported in full. For the present synthesis we additionally appraised the contemporary recommendations of the major international societies alongside the 5th edition WHO classification [6] and identified further primary studies by targeted literature searching and by hand-searching the reference lists of retrieved articles. Evidence was appraised narratively, with priority given to national registry data and large multicentre cohorts over single-centre retrospective series. No new patient-level data were generated or analysed for this manuscript.

### 2.2. Derivation of the Risk Strata and Follow-Up Intervals

The four risk strata were not derived from a formal Delphi process, nor were they mapped directly to specific hazard or odds ratios. They were generated by consensus within the gynaecological oncology multidisciplinary team at Barts Health NHS Trust, informed by the direction and magnitude of the pooled recurrence estimates in our meta-analysis [13] and by the determinants summarised in Table 1. Factors reproducibly associated with a materially higher recurrence risk across sources (extra-ovarian disease, micropapillary or cribriform architecture, and previous recurrence) were used to define the highest stratum; factors carrying an intermediate excess risk (cystectomy, intra-operative spillage or pre-operative rupture, and mucinous intraepithelial carcinoma) defined the moderate stratum; and adequately staged Stage 1a disease without adverse features defined the lowest stratum. Surveillance intervals and total duration of follow-up were then set pragmatically, anchored to the reported timing of recurrence (37% within 2 years, 31% between years 2 and 5, 32% beyond year 5 and approximately 10% beyond 10 years) [13,14] and to the follow-up schedules recommended by international societies, and agreed on by consensus. The framework is therefore a single-institution, consensus-derived proposal that has not yet been externally validated; its limitations are set out in the relevant section.

## 3. Results

### 3.1. Scope of Follow-Up

**Figure 1:**
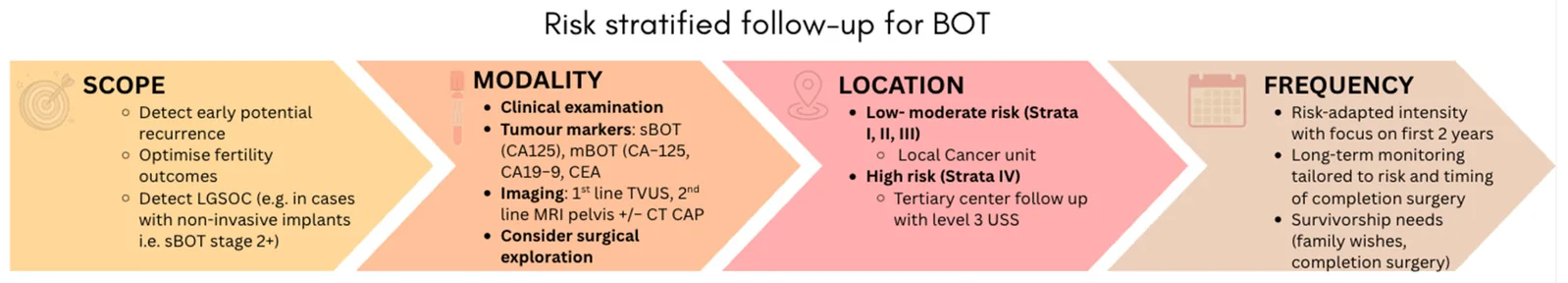
Follow-up parameters for a risk-adapted framework.

Available evidence defines three distinct targets for BOT follow-up (Figure 1): to detect malignant transformation; to detect and act on a potential recurrence as early as possible; and to improve and optimise fertility outcomes.

For Stage I tumours (~70% of BOTs at presentation), the aim of follow-up is to detect recurrence and often re-offer FSS, if fertility preservation remains a priority for the patient [30]. For Stage II–III—with non-invasive implants (likely sBOT)—the primary follow-up aim should be to detect patients that can recur as LGSOC [31,32,33]. Patients who have yet to complete their family should be actively encouraged to consult fertility experts if there are any concerns related to natural conception.

### 3.2. Available Tools (Clinical Examination, Imaging Modality and TMs)

Tools to deliver follow-up often include clinical examination, serum tumour markers (CA125, CEA, and CA 19-9), imaging modality including Uss (sonographer or doctor-led), CT of chest/abdomen/pelvis, MRI pelvis, and second-look diagnostic surgery [30,31,33].

Transvaginal ultrasound is the preferred modality for surveillance, particularly following fertility-sparing surgery. If there is no residual ovary, then USS is limited but not precluded, especially if done by an experienced clinician or trained sonographer. Cross-sectional imaging (CT or MRI) should be reserved for suspicious clinical findings, rising tumour markers, or suspected extra-ovarian recurrence [30]. To make surveillance reproducible outside tertiary centres, ultrasound findings should be reported using a standardised risk-stratification system rather than the subjective descriptor “suspicious”. IOTA ADNEX model [34], or the O-RADS ultrasound system [35], and O-RADS MRI [36] are used globally. Anchoring surveillance imaging to these systems allows the same threshold for escalation to be applied in a local gynaecology unit and in a tertiary centre, and therefore directly supports the decentralised model of care proposed below.

Serum tumour markers may be used selectively. CA-125 is certainly useful if elevated pre-operatively [33]. It also may help detect malignant transformation, especially in extra-ovarian disease. CA-125 is elevated in approximately 24–61% of BOT cases but has limited sensitivity compared with invasive ovarian cancer [1]. Other markers such as CEA or CA19-9 may be considered in mucinous BOTs when elevated at diagnosis [31]. Several caveats are important. Given this limited sensitivity, a normal CA-125 does not exclude recurrence; tumour markers should therefore never be used in isolation, nor as the sole basis for reassurance or discharge. Their principal value is in women whose markers were elevated pre-operatively, in whom serial measurement can be interpreted against a known baseline. HE4 and the ROMA algorithm have not been validated in BOTs and we do not recommend them for BOT surveillance. Anti-Müllerian hormone (AMH) has no role in oncological surveillance and does not predict recurrence, but it may be helpful in counselling women who have undergone, or are being considered for, repeated cystectomy or other fertility-sparing procedures, where it provides an objective measure of ovarian reserve to inform the timing of conception or of referral for fertility preservation. Where used for this purpose, AMH should be requested and interpreted with reproductive medicine input, and a value obtained before repeating conservative surgery is considerably more informative than a single post-operative measurement. Finally, artificial-intelligence-assisted image interpretation is developing rapidly in gynaecology and has been shown to improve diagnostic consistency in other gynaecological imaging settings [37]. Its application to adnexal ultrasound may, in future, reduce inter-operator variability in decentralised surveillance, although it is not yet ready for routine clinical use.

### 3.3. Risk Strata

**Figure 2:**
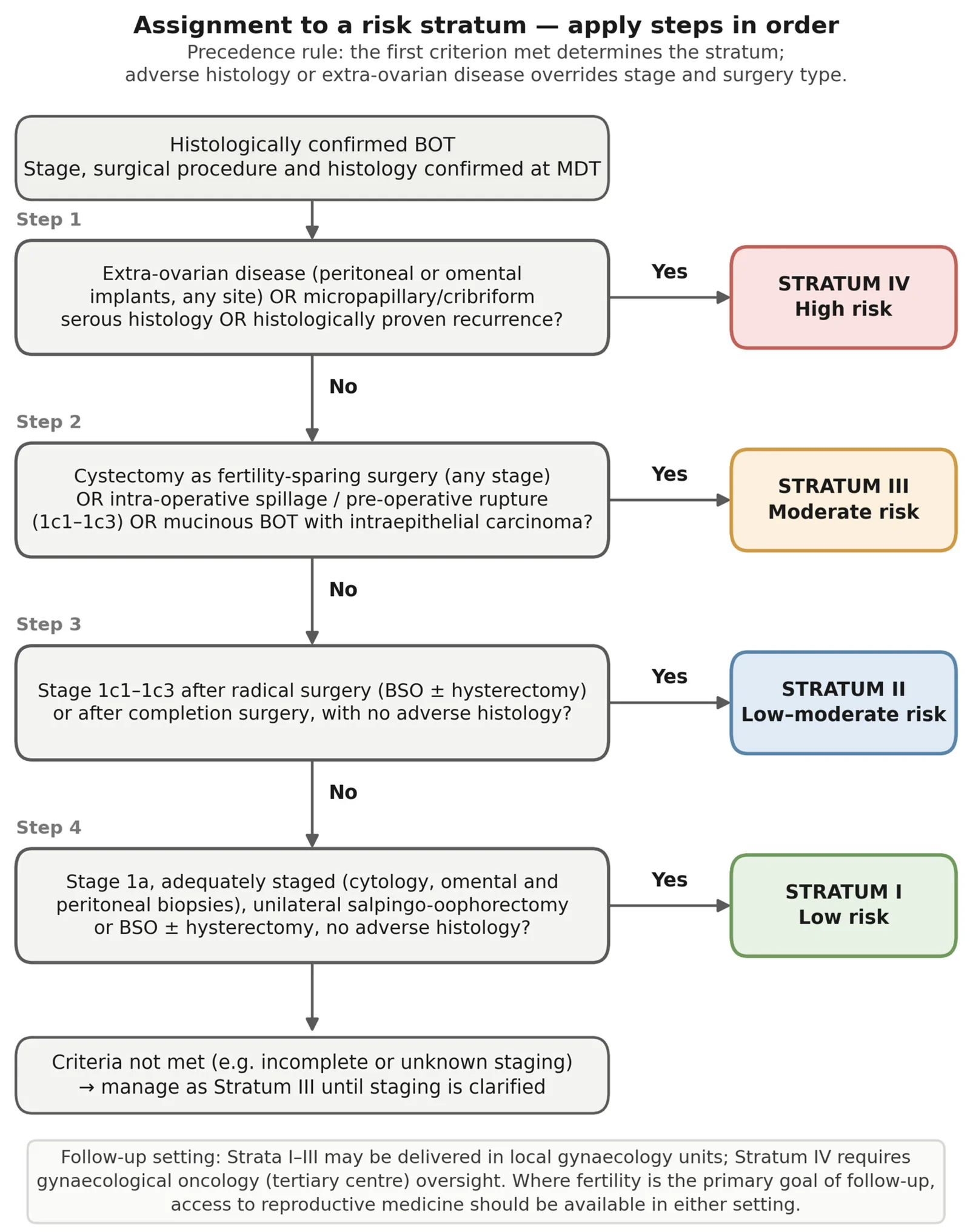
Decision algorithm for allocation to a risk stratum, with explicit precedence rules. The steps are applied in order and the first criterion met determines the stratum. BOT, borderline ovarian tumour; BSO, bilateral salpingo-oophorectomy; MDT, multidisciplinary team.

Because stage, type of surgery, histology and spillage interact, an individual patient may meet the descriptors of more than one stratum. The strata are therefore applied in a fixed order of precedence, and the highest stratum for which a patient qualifies determines her surveillance. Adverse serous histology (micropapillary or cribriform architecture), extra-ovarian disease at any site, or histologically proven recurrence always assign a patient to Stratum IV, irrespective of FIGO stage or the type of surgery performed. In the absence of those features, cystectomy, intra-operative spillage or pre-operative rupture, or mucinous intraepithelial carcinoma are assigned to Stratum III. Where staging is incomplete, or the operative record is insufficient to apply the algorithm, patients should be managed as Stratum III until staging has been clarified. Figure 2 sets out the algorithm in full, Table 2 shows the corresponding surveillance schedules and total duration of follow-up, and Table 3 shows three worked examples.

Risk Strata I (low risk): Women with Stage 1a sBOTs/mBOTs and no adverse histology features (cribriform/micropapillary architecture), who underwent adequate (cytology, omental and peritoneal biopsies) FSS, in the form of unilateral salpingo-oophorectomy (USO), or those who underwent bilateral salpingo-oophorectomy (BSO) +/− hysterectomy (radical surgery).

Risk Strata II (low–moderate risk): Women with Stage 1c1–1C3 who underwent radical surgery (BSO with/without hysterectomy including omental and peritoneal biopsies) and no adverse histology features (cribriform/micropapillary architecture), or those who underwent completion surgery for Stage 1c1–1C3.

Risk Strata III (moderate risk): Women with sBOTs/mBOTs who underwent FSS in the form of cystectomy with omental +/− peritoneal biopsies for Stage 1a–1c3. FSS in the form of cystectomy should be regarded as Stage 1c1 given the high risk of spillage; specimen handling and the route of retrieval are relevant here, since techniques that reduce uncontrolled rupture during extraction may limit iatrogenic upstaging [38]. Women who underwent USO with spillage or pre-op rupture (1c1–1c3) with no adverse histology features (cribriform/micropapillary); women with mBOTs and intraepithelial carcinoma.

Risk Strata IV (high risk): Women who underwent any form of surgery with extra-ovarian disease (including omental or peritoneal implants), or women with sBOTs and cribriform/micropapillary architecture. Women with recurrent disease.

### 3.4. Location of Follow-Up Delivery

Patients who fall into Risk Strata I, II and III are safe to be discharged to, or to continue to be followed-up in, local gynaecology units, whereas patients who fall into Risk Strata IV should remain in, or be referred to, tertiary gynaecological oncology centres. In either case, if fertility remains the primary goal of follow-up, local units or tertiary gynaecological oncology centres should have access to fertility experts to support the further interventions required to achieve family plans. The case for decentralised delivery is organisational rather than oncological: Strata I–III require only modalities that are universally available in a gynaecology unit (telephone review, transvaginal ultrasound and tumour markers). The determinants that drive allocation, including completeness of staging, specimen handling and extraction technique, and final histology are established at the index operation rather than during follow-up [38]. Standardised ultrasound reporting [34,35,36] is the principal prerequisite for delivering this safely outside a tertiary centre.

### 3.5. The Role of Gold-Standard Documentation

To facilitate risk stratification, BOT cases discussed in a multidisciplinary (MDT) setting should maintain gold-standard documentation. This includes clinical stage and surgical modality (Radical = BSO+/− hysterectomy with omental +/− peritoneal biopsy, FSS c USO/cystectomy and omental +/− peritoneal biopsies) along with a clearly stated comment on adequacy of staging (operation title and findings). Histological summary should comment on the presence of adverse features, including cribriform, micropapillary, implants and extra-ovarian confirmation or recurrent disease which is histologically proven.

### 3.6. Framework Proposal

**Figure 3:**
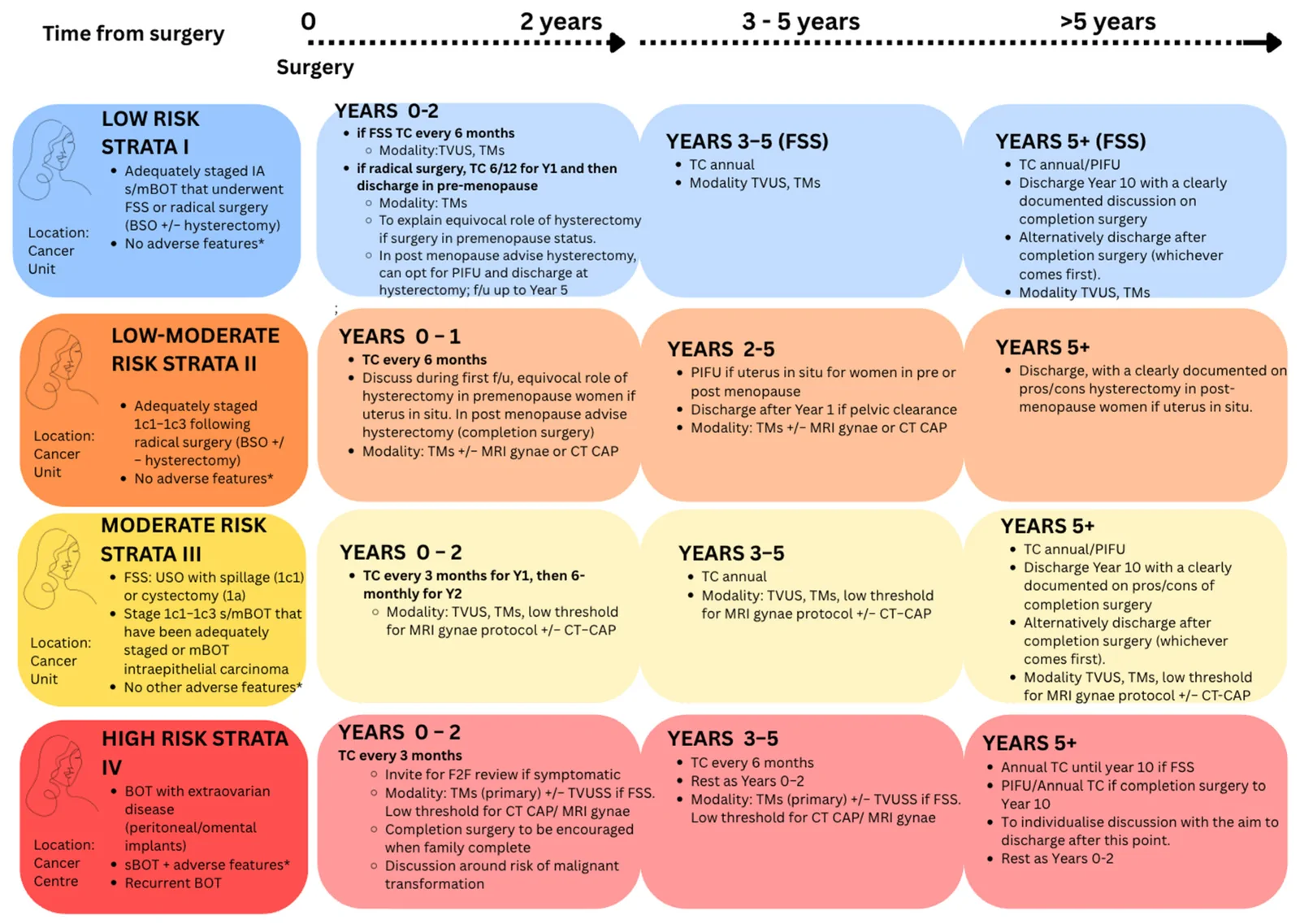
Risk-adapted follow-up surveillance framework; * adverse histological features like micropapillary/cribriform architecture.

Based on the above sections, Figure 3 summarises the follow-up framework. This includes the follow-up frequency, modality and the location of follow-up. A Telephone Call (TC) should include a targeted review of red-flag symptoms such as persistent abdominal bloating/distension, unexplained change in bladder or bowel habits, early satiety, pelvic or abdominal pain with no other obvious aetiology, weight loss or loss of appetite, investigations, and fertility aspirations, as well as the role of completion surgery, including hysterectomy [26]. A face-to-face review should be indicated in cases that require physical examination or to discuss a high likelihood of recurrence and further course of action (Strata IV). A patient-initiated follow-up (PIFU) should be part of the follow-up framework to minimise unnecessary appointments (Figure 3).

PIFU should not be offered as an open-ended discharge. It requires a named point of contact with a guaranteed response time and a written, plain-language list of the escalation triggers described above. PIFU also necessitates a defined route back into the service that does not require a fresh referral from primary care and a fixed date at which the arrangement is reviewed. Escalation should be prompted by any new or persistent red-flag symptom, by a new abdominal or pelvic mass, or by new difficulty conceiving where fertility is a goal of follow-up. We would not recommend PIFU as the default for women with limited health literacy, for those who require an interpreter, for women without reliable telephone or internet access, or for those who have expressed high levels of health anxiety, because self-directed follow-up can widen inequity by transferring the burden of vigilance to the patient [39,40]. In these circumstances clinician-initiated follow-up should be retained and the reason documented.

We would emphasise that the intervals proposed here are pragmatic and consensus-based. No randomised or prospective comparative evidence demonstrates that more intensive surveillance improves overall or disease-specific survival in BOTs, and the excellent prognosis of these tumours makes such a benefit inherently difficult to demonstrate. The value of the framework is therefore resource-rational and safety-oriented. It aims to standardise practice, to avoid both over-investigation of low-risk women and under-recognition of high-risk features, and to state explicitly where follow-up should take place and when it should stop, and not to claim a survival advantage.

Selection of imaging modality should be guided by the presence or absence of residual ovarian tissue and by risk stratum. Where an ovary remains in situ, transvaginal ultrasound (TVUSS) is the first-line modality. In women with an ovary in situ who fall into Risk Strata III–IV, a low threshold should be maintained for cross-sectional imaging with MRI (with or without CT of chest, abdomen and pelvis [CT CAP]), although ultrasound may still be performed as the initial assessment. Where no ovarian tissue remains in situ, serum biomarkers should be the first-line investigation; if imaging is subsequently required, the choice between CT CAP and MRI should be directed by whether distant or pelvic recurrence, respectively, is suspected. Cross-sectional imaging includes an MRI gynaecological protocol with IV contrast if the abnormality is located within ovarian tissue or CT CAP if distant recurrence is suspected. Serum biomarkers (Tumour Markers—TMs) should include Ca125 for sBOTs or Ca125/Ca199/CEA for mBOTs. Their role is mostly established when elevated preoperatively, or in the presence of extra-ovarian disease, in which case a rising value may indicate malignant transformation [31]. This recommendation requires one qualification. In women who have undergone radical surgery with no residual ovarian tissue and whose pre-operative tumour markers were normal, serial marker measurement may have low diagnostic utility. In such cases, there is no informative baseline against which to interpret a result, and the negative predictive value of a normal CA-125 in BOTs is poor. In this specific group we do not recommend routine serial tumour markers. Surveillance should instead be symptom-directed, with a low threshold for cross-sectional imaging (CT CAP, or MRI where pelvic recurrence is suspected) should red-flag symptoms develop. Serial markers should be reserved for women in whom a marker was elevated pre-operatively, or in whom extra-ovarian disease was documented.

## 4. Discussion

We propose a novel follow-up framework tailored to risk of recurrence, based on known histological adverse features and different surgical approaches, integrating individualised fertility aspirations.

### 4.1. Interpretation

The development of an internationally adaptable follow-up framework is essential to standardise clinical practice. Such an approach minimises unnecessary resource utilisation, enables individual units to establish services safely, and ensures that potentially high-risk cases are appropriately identified and referred to tertiary centres. It also supports timely detection of recurrence and provides reassurance to non-specialists, allowing them to manage BOTs safely within a protocol-driven framework. At the same time, we emphasise the importance of individualisation, both in determining the scope of follow-up and in implementing risk-adapted surveillance strategies.

Clinical services inherently rely on a combination of senior and junior clinicians. The implementation of a robust and clearly defined protocol enables junior clinicians to understand the underlying rationale and deliver safe care in this relatively specialised field, where senior presence in outpatient settings may be limited. This approach promotes more efficient resource allocation, allowing district-level units to safely manage follow-up and appropriately signpost women with BOTs. Within the NHS, this framework represents a cost-effective strategy by reducing the burden on tertiary centres, while maintaining patient safety as the primary priority. Although the NHS 10-year plan supports a centralised model of care, it also advocates for a shift towards community-based services. This framework therefore enables safe follow-up of BOTs within local gynaecological units or community-based gynaecological clinics, provided that clear referral pathways to tertiary centres and fertility specialists are maintained.

Unnecessary follow-up appointments, imaging, and investigations should be avoided, as they may inadvertently increase patient anxiety without providing proven screening value or clinical benefit. This framework also supports the use of patient-initiated follow-up (PIFU), an approach that is increasingly recognised and widely accepted internationally in the management of low-risk tumours [39,40].

### 4.2. Comparison with Previous Studies and Guidelines

Our framework is consistent in direction with previous work but differs in granularity. International guidance converges on the principle of prolonged follow-up with ultrasound as the primary modality, yet stops short of specifying intensity by risk group. Current recommendations advise clinical review with ultrasound without stratifying the interval by surgical approach or histology, and none states a total duration of follow-up or an explicit threshold for discharge [9,12]. The large cohort studies that underpin those recommendations—the AGO ROBOT cohort [23], the Danish nationwide registry study [24] and the Trillsch series [15,16,19]—identify the same four determinants that we have used to construct the strata (fertility-sparing surgery, incomplete staging, residual disease and advanced stage). That concordance supports the internal validity of the stratification, even though the strata themselves have not yet been tested prospectively.

Our allocation of cystectomy to a higher stratum is congruent with Morice et al., who identified cystectomy in mucinous BOTs and micropapillary architecture in serous BOTs among the factors associated with invasive recurrence [30], and with the pooled estimates in our own meta-analysis [13]. Conversely, our decision not to escalate surveillance for microinvasion alone follows the consistent observation across series that microinvasion does not alter outcome in Stage I disease [4,10]. The same logic of translating reproducible recurrence determinants into a graduated surveillance schedule, rather than applying a uniform protocol, has been advanced for other fertility-preserving gynaecological procedures [25]. The principal novelty of the present work is therefore not the identification of risk factors, which is well established, but their translation into an operational schedule that also specifies where care should be delivered and when it should stop.

### 4.3. Strengths and Limitations

The strengths of this work are that it is grounded in a previously published systematic review and meta-analysis by the same group, that it is explicit about precedence where risk factors co-exist, and that it states a total duration of follow-up for each stratum rather than leaving discharge to individual discretion. Several limitations should nonetheless be acknowledged. First, this is a narrative expert synthesis rather than a systematic review, and is therefore susceptible to selection bias in the evidence cited. Second, the strata and intervals were derived by consensus within a single tertiary centre, without a formal Delphi process or external stakeholder review; other units may reasonably arrive at different thresholds. Third, the framework has not been externally validated: we have not tested the inter-observer reproducibility of stratum allocation, nor demonstrated that it improves detection of recurrence, reduces appointments, or is acceptable to patients. Fourth, the underlying evidence is dominated by retrospective cohorts with a median follow-up of approximately five years, which is shorter than the interval over which a substantial minority of BOT recurrences arise, so risk beyond ten years is likely to be under-estimated. Fifth, the framework assumes access to good-quality transvaginal ultrasound and to a functioning gynaecological oncology MDT, which may limit transferability to health systems configured differently from the NHS. Patient-reported outcomes, anxiety and cost-effectiveness were not formally modelled, so the resource savings we anticipate remain an inference rather than a measured effect. The framework should therefore be regarded as a structured proposal to be tested, not as a validated standard of care.

## 5. Conclusions

BOTs and low-grade carcinomas appear to share a similar molecular profile. Both are commonly associated with KRAS and BRAF mutations, as well as increased expression of c-Fos, in contrast to high-grade carcinomas [1]. These tumours are thought to develop gradually from benign precursors and are characterised by relative chemoresistance. Emerging evidence suggests that the BRAF V600E mutation may represent a favourable prognostic marker, while KRAS mutations in serous BOTs (sBOTs) may be associated with extra-ovarian disease. To date, however, no additional predictive or prognostic molecular biomarkers have been consistently validated in BOTs.

As omics approaches—particularly genomics and transcriptomics—are increasingly integrated into diagnostic, prognostic, and treatment stratification, their incorporation into risk-adapted follow-up frameworks will likely become essential. Although a robust molecular classification for BOTs remains an aspirational goal, future follow-up strategies should aim to reflect emerging molecular insights. The Cancer Genome Atlas paradigm [41] continues to serve as a framework for molecular classification across multiple tumour types, with endometrial cancer representing one of the earliest examples of a disease in which management and follow-up have been informed by molecular stratification.

Several future directions follow from this work. The immediate priority is external validation. We intend to evaluate the framework prospectively within our own cohort and to invite collaborating units to apply it independently, with pre-specified endpoints of inter-observer agreement in stratum allocation, the stage and mode of detection of recurrence, the number of appointments and investigations per patient-year, and patient-reported acceptability. Because a substantial minority of recurrences arise beyond five years, a meaningful assessment of oncological safety will require a minimum of five and preferably ten years of follow-up, and registry-based collaboration is likely to be necessary to accrue sufficient events. In parallel, standardised ultrasound reporting [34,35,36] should be embedded so that surveillance findings are comparable between centres, and the developing role of artificial intelligence in gynaecological image interpretation [37] may in time reduce inter-operator variability and support the safe delivery of surveillance in non-specialist settings. Ultimately, the integration of molecular stratification with these operational and imaging developments offers the prospect of a genuinely individualised, biology-driven surveillance strategy.

Finally, the implementation of a standardised follow-up strategy may also facilitate more consistent reporting of follow-up outcomes and reduce research waste.

## Figures and Tables

**Table 1:** Clinicopathological factors consistently associated with increased risk of recurrence.

Risk Factor Category	Factor	Impact on Recurrence	Key Evidence
Surgical factors	Fertility-sparing surgery (FSS)	Increased recurrence compared with radical surgery	[15,23,24]
	Cystectomy vs. oophorectomy	Higher recurrence after cystectomy (~31–41%) than after oophorectomy (~9–13%)	[12,13]
	Incomplete surgical staging	Associated with increased recurrence risk	[9,23,24]
	Residual tumour following surgery	Independent prognostic factor for recurrence	[9,23,24]
Histopathological factors	Micropapillary/cribriform architecture in serous BOTs	Higher risk of extra-ovarian disease, recurrence and disease-related mortality	[6,17,18]
	Invasive peritoneal implants (WHO 2020: low-grade serous carcinoma)	Established risk factor for persistent or recurrent disease	[3,6,9]
	High-grade transformation of a low-grade serous neoplasm	Rare; associated with poor prognosis	[20,21,22]
Tumour-related factors	Advanced FIGO stage	Independent prognostic factor for recurrence	[23,24]
	Bilateral ovarian involvement	Associated with increased recurrence risk	[13]
	Large tumour size (>10 cm)	Associated with higher recurrence rates	[3,12,14]
Patient-related factors	Age 40 years and above	Increased recurrence risk and higher likelihood of malignant transformation	[19]
Histological subtype	Serous BOT (sBOT)	More likely to recur as peritoneal disease	[1,3]
	Mucinous BOT (mBOT)	Recurrence typically ovarian; greater risk of progression to invasive mucinous carcinoma where intraepithelial carcinoma is present and spillage occurs	[1,3]

Numbers in the “Key evidence” column refer to the reference list. BOT, borderline ovarian tumour; FIGO, International Federation of Gynecology and Obstetrics; FSS, fertility-sparing surgery; WHO, World Health Organization.

**Table 2:** Operational summary of the four risk strata: defining criteria, surveillance schedule, total duration of follow-up and care setting.

Stratum	Defining Criteria (Applied in Order of Precedence)	Surveillance Schedule and Modality	Total Duration and Discharge	Setting
I Low risk	Stage 1a sBOT/mBOT, adequately staged (cytology, omental and peritoneal biopsies), treated by USO or by BSO ± hysterectomy; no adverse histology	Years 0–2: after FSS, telephone review 6-monthly (TVUSS + TMs); after radical surgery, telephone review at 6 and 12 months (TMs). Years 3–5 (FSS): annual telephone review with TVUSS + TMs	After radical surgery, discharge at 12 months (premenopausal), or by year 5 where hysterectomy is deferred. After FSS, annual review or PIFU to year 10, or discharge after completion surgery, whichever occurs first.	Cancer unit
II Low–moderate risk	Stage 1c1–1c3 after radical surgery (BSO ± hysterectomy with omental and peritoneal biopsies) or after completion surgery; no adverse histology	Years 0–1: telephone review 6-monthly (TMs ± MRI gynaecological protocol or CT CAP). Years 2–5: PIFU where the uterus remains in situ	Discharge after year 1 where pelvic clearance is complete; otherwise PIFU to year 5, with a documented discussion of hysterectomy in postmenopausal women with a uterus in situ	Cancer unit
III Moderate risk	Cystectomy as FSS (any stage, treated as 1c1); USO with spillage or pre-operative rupture (1c1–1c3) without adverse histology; mBOT with intraepithelial carcinoma; or staging incomplete or unavailable	Years 0–2: telephone review 3-monthly in year 1, then 6-monthly in year 2 (TVUSS + TMs, low threshold for MRI gynaecological protocol ± CT CAP). Years 3–5: annual telephone review, same modalities	Annual review or PIFU from year 5; discharge at year 10 with a documented discussion of completion surgery, or after completion surgery, whichever occurs first	Cancer unit
IV High risk	Extra-ovarian disease (peritoneal or omental implants) after any surgery; sBOT with micropapillary/cribriform architecture; histologically proven recurrent disease	Years 0–2: telephone review 3-monthly, with face-to-face review if symptomatic (TMs primary ± TVUSS where an ovary remains; low threshold for CT CAP or MRI). Years 3–5: 6-monthly, otherwise as years 0–2	Annual telephone review or PIFU to year 10 (PIFU where completion surgery has been performed); thereafter individualised, with discharge considered case by case	Cancer centre (gynaecological oncology)

Strata are applied in descending order of precedence (Stratum IV first); see Figure 2. BSO, bilateral salpingo-oophorectomy; CT CAP, computed tomography of chest, abdomen and pelvis; FSS, fertility-sparing surgery; mBOT, mucinous borderline ovarian tumour; PIFU, patient-initiated follow-up; sBOT, serous borderline ovarian tumour; TMs, tumour markers; TVUSS, transvaginal ultrasound; USO, unilateral salpingo-oophorectomy. Intervals are pragmatic and consensus-based; no evidence currently demonstrates that more intensive surveillance improves survival in BOTs.

**Table 3:** Worked examples illustrating application of the precedence rules.

Clinical Vignette	Application of the Algorithm (Figure 2)	Allocated Stratum
A 29-year-old woman with a 12 cm Stage 1a mucinous BOT treated by laparoscopic cystectomy with omental and peritoneal biopsies; capsule intact; no intraepithelial carcinoma	Step 1 is not met (no extra-ovarian disease, no adverse serous histology, no recurrence). Step 2 is met: cystectomy was performed as fertility-sparing surgery and is therefore treated as Stage 1c1 irrespective of the recorded 1a stage. Allocation stops at Step 2; large tumour size and young age do not modify the stratum	Stratum III
A 46-year-old woman with a Stage 1c2 serous BOT treated by BSO and hysterectomy with omental and peritoneal biopsies; micropapillary architecture reported on final histology	Step 1 is met: micropapillary architecture is present. Adverse histology overrides both the completeness of radical surgery and the early stage, so allocation is to Stratum IV rather than to Stratum II on stage alone	Stratum IV
A 34-year-old woman with a Stage 1a serous BOT treated by USO with cytology, omental and peritoneal biopsies; no spillage; no adverse features; wishes to conceive	Steps 1–3 are not met; Step 4 is met. Allocation is to Stratum I. Because fertility-sparing surgery was performed, the FSS schedule in Table 2 applies and access to reproductive medicine should be available	Stratum I

## Data Availability

The original contributions presented in this study are included in the article. Further inquiries can be directed to the corresponding author.

## Abbreviations

The following abbreviations are used in this manuscript:

ADNEX	Assessment of Different NEoplasias in the adneXa (IOTA model)
AMH	Anti-Müllerian hormone
BOT	Borderline ovarian tumour
BSO	Bilateral salpingo-oophorectomy
CT CAP	Computed tomography of chest, abdomen and pelvis
FIGO	International Federation of Gynecology and Obstetrics
FSS	Fertility-sparing surgery
IOTA	International Ovarian Tumour Analysis
HE4	Human epididymis protein 4
LGSOC	Low-grade serous ovarian carcinoma
mBOT	Mucinous borderline ovarian tumour
MDT	Multidisciplinary Team
MRI	Magnetic resonance imaging
NHS	National Health Service
OC	Ovarian cancer
O-RADS	Ovarian-Adnexal Reporting and Data System
PIFU	Patient-initiated follow-up
ROMA	Risk of Ovarian Malignancy Algorithm
sBOT	Serous borderline ovarian tumour
TC	Telephone call
TM	Tumour marker
TVUSS	Transvaginal ultrasound
USO	Unilateral salpingo-oophorectomy
WHO	World Health Organization
